# Self-reported artificial intelligence use, knowledge, and perceptions among undergraduate medical students at a Peruvian university

**DOI:** 10.3389/frai.2026.1940222

**Published:** 2026-10-29

**Authors:** Karen Avilés-Antezana, Alonso Soto, Iván Hernández-Patiño, José M. Vela-Ruiz, Jaime Lama-Valdivia

**Affiliations:** 1 Facultad de Medicina Humana, Instituto de Investigaciones en Ciencias Biomédicas (INICIB), Universidad Ricardo Palma, Lima, Peru; 2 Instituto de Investigaciones en Ciencias Biomédicas, Universidad Ricardo Palma, Lima, Peru; 3 Hospital de Emergencias Villa El Salvador, Lima, Peru

**Keywords:** artificial intelligence, digital health, medical education, perception, technology adoption, undergraduate medical students

## Abstract

Artificial intelligence (AI) is increasingly used in medical education, yet evidence regarding its actual use, knowledge, and perceptions among undergraduate medical students in Peru remains limited. This study aimed to estimate the prevalence of self-reported AI use for academic activities and to describe patterns of use, self-reported knowledge, and perceptions among undergraduate medical students at a Peruvian university, with an exploratory assessment of associated characteristics. We conducted an observational, analytical, cross-sectional study in 2024 among students enrolled at Universidad Ricardo Palma. A total of 279 responses were received; one respondent did not provide informed consent and was excluded, leaving 278 participants for analysis. Data were collected using an adapted online questionnaire based on previously published instruments. AI use was assessed through a separate yes/no question regarding previous use of any AI tool. Overall, 257 of 278 participants (92.4%) reported previous AI use for academic activities. Most participants self-reported basic knowledge of AI principles (89.2%) and awareness of its use in medicine (95.3%). Among the predefined AI-tool categories, ChatGPT (69.1%) and Canva (19.8%) were the most frequently reported. The primary contribution of this study is descriptive, documenting the prevalence and patterns of AI use, self-reported knowledge, and perceptions in the surveyed population. Secondary exploratory regression analyses suggested associations with daily digital-device use and perceptions regarding treatment planning, but these estimates should be interpreted cautiously because only 21 participants were classified as non-users. These findings highlight the need for structured education addressing critical, ethical, and responsible AI use in undergraduate medical training.

## 1 Introduction

Artificial intelligence (AI) is increasingly being incorporated into healthcare and medical education. In clinical settings, AI has been applied to areas such as diagnostic support, medical imaging, clinical decision-making, personalized care, and healthcare system optimization. In parallel, the rapid development of generative AI tools, particularly large language models, has expanded their use among medical students for activities such as information retrieval, content generation, academic writing support, and self-directed learning. However, the growing availability of these technologies has also raised concerns regarding accuracy, transparency, bias, privacy, academic integrity, and appropriate use in educational and clinical contexts (World Health Organization, 2021; Miao et al., 2021; Gordon et al., 2024).

Recent studies suggest that medical students generally show high awareness of AI and favorable attitudes toward its incorporation into medical training. Nevertheless, reported levels of knowledge, confidence, and actual use remain heterogeneous across settings (Tolentino et al., 2024; Bisdas et al., 2021; Ahmed et al., 2022; Civaner et al., 2022; Al-Qerem et al., 2023; Ganjavi et al., 2024; Weidener and Fischer, 2024). Similar gaps between interest in AI and familiarity with its underlying concepts have also been reported among practicing physicians (Banerjee et al., 2024), underscoring that awareness, perceived knowledge, and substantive competence are not interchangeable. This distinction is important because positive perceptions toward AI do not necessarily imply frequent or appropriate use. Furthermore, the adoption of AI tools may be related to multiple factors, including digital exposure, access to technology, previous experience, self-reported knowledge, and perceived usefulness.

In Peru, evidence on AI among medical students remains limited. A previous Peruvian study conducted among medical students primarily explored perceptions toward AI and reported generally favorable attitudes toward its incorporation into medical education (Bautista Huaytalla and Flores Larzo, 2024). However, there is still limited information regarding the self-reported use of AI for academic activities and the characteristics associated with such use in Peruvian medical students. This local evidence is relevant because patterns of AI use may differ according to educational context, digital access, institutional guidance, and students’ exposure to emerging technologies.

Therefore, this study aimed to estimate the prevalence of self-reported AI use for academic activities and to describe patterns of use, self-reported knowledge, and perceptions among undergraduate medical students at a Peruvian university, with an exploratory assessment of associated characteristics.

## 2 Materials and methods

### 2.1 Study design and setting

This observational, analytical, cross-sectional study was conducted among undergraduate medical students enrolled in the School of Medicine at Universidad Ricardo Palma, Lima, Peru, during the 2024 academic year. Data collection was conducted between November and December 2024.

### 2.2 Participants, sampling and sample size

The target population comprised undergraduate medical students enrolled at the Faculty of Human Medicine of Universidad Ricardo Palma during the 2024 academic year. Participants were recruited using non-probability convenience sampling through the official WhatsApp groups of each academic year.

The minimum sample size was estimated using OpenEpi for an analytical cross-sectional study, assuming a 95% confidence level and 80% statistical power and using the study by Ahmed et al. (2022) as the reference for the calculation. The initial estimated sample size was 248 participants. An additional 10% was added to account for potential recruitment losses, resulting in a minimum target sample of 273 participants.

A total of 279 survey responses were received. One respondent did not provide informed consent and was therefore excluded before analysis. The remaining 278 participants were included in the final analytical sample.

### 2.3 Eligibility criteria

Undergraduate medical students enrolled at Universidad Ricardo Palma during the study period who voluntarily agreed to participate and provided electronic informed consent were eligible for inclusion. Respondents who did not provide informed consent were excluded. Among participants who consented to participate, no questionnaires were excluded because of missing data, resulting in a final analytical sample of 278 students.

### 2.4 Variables

The primary outcome was self-reported previous use of artificial intelligence, assessed through the question, “Have you ever used any AI tool?” with two response options (yes/no). This variable was analyzed independently from the separate questionnaire item concerning specific AI tools used.

Participants were also asked to select from a predefined list of AI tools, which included ChatGPT, Grammarly, Canva, Meta AI, Jasper AI, Copilot AI, and an option indicating that none of the listed tools was used. The latter response indicated that the participant did not use any of the tools specifically listed in the questionnaire and was not considered equivalent to reporting no previous AI use on the primary outcome question.

Other study variables included age, sex, availability of digital devices (mobile phone, tablet, laptop, and desktop computer), average daily hours of general digital-device use, monthly family income in Peruvian soles (PEN), academic year, self-reported basic knowledge of AI, awareness of AI use in medicine, sources of information about AI, and perceptions regarding the role and potential applications of AI in medicine.

### 2.5 Data collection instrument and procedure

Data were collected between November and December 2024 using a voluntary online questionnaire administered through Google Forms. The survey link was distributed by student representatives through the official WhatsApp groups corresponding to each academic year of the School of Medicine. The form was configured to allow one response per participant. The number of students who received or viewed the invitation was not recorded; therefore, a formal response rate could not be calculated.

The questionnaire was adapted from the instrument developed by Yüzbaşıoğlu (2021) and from a Peruvian adaptation previously used among medical students (Bautista Huaytalla and Flores Larzo, 2024). The questionnaire administered in the present study included sociodemographic and digital-use characteristics, self-reported knowledge of AI, sources of information about AI, specific AI tools, previous AI use, and perceptions regarding the application of AI in medicine.

The perception section consisted of 14 items. The source instrument used a five-category response format ranging from strong disagreement to strong agreement, including a “do not know” option. Before data collection, the response format was adapted to three categories: disagree, do not know, and agree. Thus, the three-category structure represented the response format administered to participants and was not generated by *post hoc* collapsing of responses during statistical analysis.

The item on basic AI knowledge asked participants whether they considered themselves to have basic knowledge of the principles underlying AI; therefore, this variable represents self-reported rather than objectively assessed knowledge. Daily device-use time referred to general daily use of digital devices and was not restricted to academic or AI-related activities. Monthly family income was reported directly in Peruvian soles.

### 2.6 Validity and reliability of the instrument

The perception section comprised 14 ordinal items. Because the response format of the source instrument was adapted from five to three categories before data collection, the psychometric properties of the adapted version were reassessed in the study sample. Internal consistency was high (Cronbach’s *α* = 0.8699). Sampling adequacy was satisfactory [Kaiser–Meyer–Olkin (KMO) = 0.8724], and Bartlett’s test of sphericity was statistically significant (*χ*^2^ = 1540.81; df = 91; *p* < 0.001).

Exploratory factor analysis was conducted using a polychoric correlation matrix because the perception items were ordinal. Iterated principal-factor extraction was used. The number of factors retained was determined based on eigenvalues greater than 1, visual inspection of the scree plot, and interpretability of the factor solution. Both the Kaiser criterion and the scree plot supported retention of two factors. Because the latent dimensions were expected to be correlated, an oblique Promax rotation was applied.

The resulting two-factor structure comprised a factor predominantly reflecting clinical applications of AI and a second factor reflecting general attitudes and educational integration of AI. The correlation between the two factors was 0.587. McDonald’s omega, estimated from the polychoric two-factor solution, was 0.933, indicating high reliability. Factor loadings and uniqueness estimates are presented (Supplementary Table S1).

### 2.7 Statistical analysis

Data were analyzed using Stata version 16.1. Categorical variables were summarized using absolute frequencies and percentages. Quantitative variables were described using medians and interquartile ranges because their distributions were non-normal or asymmetric.

The primary outcome was self-reported previous use of artificial intelligence, analyzed as a binary variable (yes/no). Exploratory bivariate analyses were performed using Poisson regression with robust variance to estimate crude prevalence ratios (cPRs) and their corresponding 95% confidence intervals (CIs). Continuous variables were entered into the models as continuous predictors. For family income, the prevalence ratio was estimated per PEN 1,000 increase to improve interpretability. Categorical variables were entered using indicator variables, with one category specified as the reference. For variables with more than two categories, an overall *p* value was obtained using post-estimation Wald tests.

Specific AI-tool categories were not included as explanatory variables in the regression analyses because they were conceptually linked to the primary outcome of AI use. Sources of information were also excluded from multivariable modeling because one category contained no participants in the non-user group, resulting in unstable global robust Wald estimation.

Given the high prevalence of the outcome and the relatively small number of participants who reported no previous AI use, the multivariable analysis was intentionally restricted to a parsimonious exploratory model to reduce the risk of overfitting and sparse-data bias. Because only 21 participants were classified as non-users, we limited the number of parameters included in the adjusted model. Candidate variables were evaluated considering the bivariate findings, their conceptual relationship with AI use, and changes in the estimates across progressively simplified models. Age and tablet availability were not retained because their exclusion produced little change in the estimates for daily device use and treatment-planning perception.

The final multivariable Poisson regression model with robust variance included daily digital-device use and perception regarding the usefulness of AI for treatment planning. Adjusted prevalence ratios (aPRs), 95% CIs, category-specific *p* values, and the overall *p* value for the perception variable were reported. Statistical significance was defined as a two-sided *p* value <0.05. Because these analyses were exploratory, findings were interpreted cautiously and emphasis was placed on effect estimates and confidence intervals rather than statistical significance alone. To assess the stability of the final estimates in the presence of sparse outcome categories, a non-parametric bootstrap sensitivity analysis with 5,000 resamples was additionally performed, and bootstrap 95% CIs were compared with those from the robust Poisson model.

### 2.8 Ethical considerations

The study was approved by the Institutional Research Ethics Committee of the Faculty of Human Medicine at Universidad Ricardo Palma (approval code PG 2152024-A). Participation was voluntary, and electronic informed consent was obtained before completion of the questionnaire. Participants who did not provide consent were not included in the study. Data were collected anonymously and used exclusively for research purposes, with measures implemented to protect participant confidentiality and privacy. The study was conducted in accordance with the ethical principles of the Declaration of Helsinki and applicable institutional requirements.

## 3 Results

### 3.1 Participant characteristics

A total of 278 medical students were included in the analysis. The median age was 22 years (IQR: 19–24), and 167 participants (60.07%) were female. Mobile phones were the most commonly available digital device (97.84%), followed by laptops (83.81%), tablets (52.16%), and desktop computers (34.53%). Participants reported a median of 6 h (IQR: 4–8) of daily digital-device use, while the median monthly family income was PEN 5,000 (IQR: 3,000–7,500). Students from all six academic years participated, with sixth-year students representing the largest group (29.14%) (Table 1).

**Table 1:** Participant characteristics and artificial intelligence tools used.

Characteristic	*n*	%
Sex		
Male	111	39.93
Female	167	60.07
Age (IQR)	22	19–24
Available devices*		
Mobile phone	272	97.84
Tablet	145	52.16
Laptop	233	83.81
Desktop computer	96	34.53
Self-reported AI use		
No	21	7.55
Yes	257	92.45
Daily hours of digital device use, median (IQR)	6	4–8
Family income (PEN), median (IQR)	5,000	3,000–7,500
Academic year		
First year	39	14.03
Second year	43	15.47
Third year	26	9.35
Fourth year	50	17.99
Fifth year	39	14.03
Sixth year	81	29.14
Reported AI tool		
None of the listed AI tools	10	3.60
Grammarly	5	1.80
Canva	55	19.78
Jasper AI	0	0
CoPilot AI	6	2.16
Meta AI	10	3.60
ChatGPT	192	69.06

IQR, interquartile range; AI, artificial intelligence. *Participants could report more than one available device; therefore, percentages do not sum to 100%.

Overall, 257 of 278 participants (92.45%) reported having previously used AI, whereas 21 (7.55%) reported no previous AI use. Among the predefined AI-tool categories, ChatGPT was the most frequently reported (69.06%), followed by Canva (19.78%). Other reported tools included Meta AI (3.60%), Copilot AI (2.16%), and Grammarly (1.80%). Ten participants (3.60%) reported using none of the AI tools specifically listed in the questionnaire (Table 1).

### 3.2 Self-reported AI knowledge and perceptions

Most participants (89.21%) self-reported having basic knowledge of AI, and 95.32% reported awareness of AI use in medicine. Social media was the most frequently reported source of information about AI (39.21%), followed by friends or family (29.14%), university readings (20.14%), and newspapers or journals (7.19%) (Table 2).

**Table 2:** Knowledge and perceptions regarding artificial intelligence in medicine.

Items	Response category	*n*	%
Self-reported basic AI knowledge			
No		30	10.79
Yes		248	89.21
Awareness of AI use in medicine			
No		13	4.68
Yes		265	95.32
Sources of information			
None		12	4.32
Friends or family		81	29.14
Newspaper or journals		20	7.19
University readings		56	20.14
Social media		109	39.21
Perceptions of AI in medicine			
AI will lead to major advances in medicine	Disagree	21	7.55
	Do not know	29	10.43
	Agree	228	82.01
AI will replace physicians in the future	Disagree	194	69.78
	Do not know	45	16.19
	Agree	39	14.03
AI can be used as a definitive diagnostic tool	Disagree	144	51.80
	Do not know	46	16.55
	Agree	88	31.65
AI can be used as a prognostic tool	Disagree	72	25.90
	Do not know	65	23.38
	Agree	141	50.72
AI can be used for treatment planning	Disagree	47	16.91
	Do not know	68	24.46
	Agree	163	58.63
AI can be used for treatment quality control	Disagree	62	22.30
	Do not know	60	21.58
	Agree	156	56.12
AI applications should be part of undergraduate training	Disagree	48	17.27
	Do not know	67	24.10
	Agree	163	58.63
AI applications should be part of postgraduate training	Disagree	47	16.91
	Do not know	71	25.54
	Agree	160	57.55
The use of AI in medicine is exciting	Disagree	26	9.35
	Do not know	50	17.99
	Agree	202	72.66
AI can be used for radiographic diagnosis	Disagree	51	18.35
	Do not know	69	24.82
	Agree	158	56.83
AI can be used for lesion diagnosis	Disagree	73	26.26
	Do not know	67	24.10
	Agree	138	49.64
AI can be used for radiographic diagnosis of respiratory pathologies	Disagree	65	23.38
	Do not know	75	26.98
	Agree	138	49.64
AI can be used for radiographic diagnosis of intestinal obstruction	Disagree	63	22.66
	Do not know	81	29.14
	Agree	134	48.20
AI can be used in forensic medicine	Disagree	71	25.54
	Do not know	86	30.94
	Agree	121	43.53

AI, artificial intelligence.

Overall, participants expressed generally favorable perceptions toward several potential applications of AI in medicine. Most agreed that AI would lead to major advances in medicine (82.01%), while 72.66% considered the use of AI in medicine exciting. More than half agreed that AI could be used for treatment planning (58.63%) and treatment quality control (56.12%), and similar proportions supported the inclusion of AI applications in undergraduate (58.63%) and postgraduate medical training (57.55%). In contrast, 69.78% disagreed that AI would replace physicians in the future, and 51.80% disagreed that AI could be used as a definitive diagnostic tool (Table 2).

### 3.3 Exploratory bivariate analysis

Because only 21 participants were classified as non-users, all regression estimates should be interpreted as exploratory and potentially imprecise, particularly for categories with small cell counts. Accordingly, emphasis was placed on effect estimates and confidence intervals rather than statistical significance alone.

In exploratory bivariate Poisson regression analyses with robust variance, greater daily digital-device use was associated with self-reported AI use. For each additional hour of daily device use, the prevalence of self-reported AI use was approximately 1% higher (cPR = 1.01; 95% CI: 1.00–1.02; *p* = 0.027). No statistically significant associations were observed for age (*p* = 0.062), family income (*p* = 0.900), sex (*p* = 0.251), academic year (overall *p* = 0.788), self-reported basic AI knowledge (*p* = 0.329), or awareness of AI use in medicine (*p* = 0.210). None of the individual device-availability variables reached conventional statistical significance (Table 3).

**Table 3:** Exploratory bivariate analysis of characteristics associated with self-reported artificial intelligence use.

Variable	Response category	No AI use (*n* = 21)	AI use (*n* = 257)	cPR (95% CI)	*p* value	Overall *p* value
Daily digital-device use, hours, median (IQR)		5 (3–6)	6 (4–8)	1.01 (1.00–1.02)	0.027	
Age (years), median (IQR)		21 (18–23)	22 (20–24)	1.00 (1.00–1.01)	0.062	
Family income (PEN), median (IQR)		4,000 (2,000–10,000)	5,000 (3,300–7,000)	1.000 (0.997–1.003)	0.900	
Sex						
Male		11 (9.91%)	100 (90.09%)	Ref.		0.251
Female		10 (5.99%)	157 (94.01%)	1.04 (0.97–1.12)	0.251	
Digital device availability						
Mobile phone		19 (6.99%)	253 (93.10%)	1.40 (0.79–2.46)	0.250	
Tablet		15 (10.34%)	130 (89.66%)	0.94 (0.88–1.00)	0.064	
Laptop		16 (6.87%)	217 (93.13%)	1.05 (0.94–1.17)	0.403	
Desktop computer		7 (7.29%)	89 (92.71%)	1.00 (0.94–1.08)	0.904	
Academic year						
First year		5 (12.82%)	34 (87.18%)	Ref.		0.788
Second year		3 (6.98%)	40 (93.02%)	1.07 (0.92–1.23)	0.383	
Third year		3 (11.54%)	23 (88.46%)	1.01 (0.84–1.22)	0.876	
Fourth year		3 (6.00%)	47 (94.00%)	1.08 (0.94–1.24)	0.290	
Fifth year		3 (7.69%)	36 (92.31%)	1.06 (0.91–1.23)	0.458	
Sixth year		4 (4.94%)	77 (95.06%)	1.09 (0.96–1.24)	0.193	
Self-reported basic AI knowledge						
No		4 (13.33%)	26 (86.67%)	Ref.		0.329
Yes		17 (6.85%)	231 (93.15%)	1.07 (0.93–1.24)	0.329	
Awareness of AI use in medicine						
No		3 (23.08%)	10 (76.92%)	Ref.		0.210
Yes		18 (6.79%)	247 (93.21%)	1.21 (0.90–1.64)	0.210	
Perceptions regarding AI in medicine						
AI will lead to major advances in medicine	Disagree	1 (4.76%)	20 (95.24%)	Ref.		0.118
	Do not know	7 (24.14%)	22 (75.86%)	0.80 (0.63–1.00)	0.049	
	Agree	13 (5.70%)	215 (94.30%)	0.99 (0.90–1.10)	0.847	
AI will replace physicians in the future	Disagree	13 (6.70%)	181 (93.30%)	Ref.		0.415
	Do not know	6 (13.33%)	39 (86.67%)	0.91 (0.80–1.05)	0.193	
	Agree	2 (5.13%)	37 (94.87%)	0.98 (0.91–1.07)	0.690	
AI can be used as a definitive diagnostic tool	Disagree	12 (8.33%)	132 (91.67%)	Ref.		0.327
	Do not know	5 (10.87%)	41 (89.13%)	0.97 (0.87–1.09)	0.625	
	Agree	4 (4.55%)	84 (95.45%)	1.04 (0.97–1.11)	0.238	
AI can be used as a prognostic tool	Disagree	7 (9.72%)	65 (90.28%)	Ref.		0.259
	Do not know	7 (10.77%)	58 (89.23%)	0.99 (0.88–1.11)	0.841	
	Agree	7 (4.96%)	134 (95.04%)	1.05 (0.97–1.15)	0.235	
AI can be used for treatment planning	Disagree	7 (14.89%)	40 (85.11%)	Ref.		0.033
	Do not know	8 (11.76%)	60 (88.24%)	1.04 (0.89–1.20)	0.633	
	Agree	6 (3.68%)	157 (96.32%)	1.13 (1.00–1.28)	0.050	
AI can be used for treatment quality control	Disagree	5 (8.06%)	57 (91.94%)	Ref.		0.099
	Do not know	9 (15.00%)	51 (85.00%)	0.92 (0.81–1.05)	0.236	
	Agree	7 (4.49%)	149 (95.51%)	1.04 (0.96–1.13)	0.358	
AI applications should be part of undergraduate training	Disagree	4 (8.33%)	44 (91.67%)	Ref.		0.326
	Do not know	8 (11.94%)	59 (88.06%)	0.96 (0.85–1.09)	0.522	
	Agree	9 (5.52%)	154 (94.48%)	1.03 (0.94–1.13)	0.525	
AI applications should be part of postgraduate training	Disagree	5 (10.64%)	42 (89.36%)	Ref.		0.401
	Do not know	7 (9.86%)	64 (90.14%)	1.01 (0.89–1.14)	0.892	
	Agree	9 (5.62%)	151 (94.38%)	1.06 (0.95–1.17)	0.312	
The use of AI in medicine is exciting	Disagree	5 (19.23%)	21 (80.77%)	Ref.		0.052
	Do not know	7 (14.00%)	43 (86.00%)	1.06 (0.86–1.33)	0.574	
	Agree	9 (4.46%)	193 (95.54%)	1.18 (0.98–1.43)	0.084	
AI can be used for radiographic diagnosis	Disagree	5 (9.80%)	46 (90.20%)	Ref.		0.105
	Do not know	9 (13.04%)	60 (86.96%)	0.96 (0.85–1.10)	0.578	
	Agree	7 (4.43%)	151 (95.57%)	1.06 (0.96–1.17)	0.241	
AI can be used for lesion diagnosis	Disagree	7 (9.59%)	66 (90.41%)	Ref.		0.132
	Do not know	8 (11.94%)	59 (88.06%)	0.98 (0.87–1.09)	0.656	
	Agree	6 (4.35%)	132 (95.65%)	1.06 (0.97–1.15)	0.183	
AI can be used for radiographic diagnosis of respiratory pathologies	Disagree	6 (9.23%)	59 (90.77%)	Ref.		0.296
	Do not know	8 (10.67%)	67 (89.33%)	0.98 (0.88–1.10)	0.777	
	Agree	7 (5.07%)	131 (94.93%)	1.05 (0.96–1.14)	0.311	
AI can be used for radiographic diagnosis of intestinal obstruction	Disagree	7 (11.11%)	56 (88.89%)	Ref.		0.168
	Do not know	8 (9.88%)	73 (90.12%)	1.01 (0.91–1.14)	0.812	
	Agree	6 (4.48%)	128 (95.52%)	1.07 (0.98–1.18)	0.137	
AI can be used in forensic medicine	Disagree	7 (9.86%)	64 (90.14%)	Ref.		0.139
	Do not know	9 (10.47%)	77 (89.53%)	0.99 (0.89–1.10)	0.900	
	Agree	5 (4.13%)	116 (95.87%)	1.06 (0.98–1.16)	0.158	

Data are presented as *n* (%) or median (IQR), as appropriate. Percentages in the No AI use and AI use columns are calculated within each category. cPR, crude prevalence ratio; CI, confidence interval; AI, artificial intelligence; IQR, interquartile range; PEN, Peruvian soles; Ref., reference category. Crude prevalence ratios, 95% confidence intervals, and category-specific p values were estimated using Poisson regression with robust variance. Overall *p* values for categorical variables with more than two categories were obtained using post-estimation Wald tests. Analyses were exploratory. Specific AI-tool categories and sources of information were not included in this regression table because the former was conceptually linked to the outcome and the latter contained a zero cell that resulted in unstable global robust Wald estimation. For family income, the crude prevalence ratio was estimated per PEN 1,000 increase to improve interpretability.

Among the perception items, responses regarding the potential use of AI for treatment planning showed an overall association with self-reported AI use (overall *p* = 0.033). Compared with participants who disagreed, those who agreed had a cPR of 1.13 (95% CI: 1.00–1.28; *p* = 0.050), whereas those who responded “do not know” had a cPR of 1.04 (95% CI: 0.89–1.20; *p* = 0.633). No other perception item showed a statistically significant overall association at the 0.05 level (Table 3).

### 3.4 Exploratory multivariable analysis

In the exploratory multivariable Poisson regression model, daily digital-device use remained associated with self-reported AI use (aPR = 1.01; 95% CI: 1.00–1.02; *p* = 0.020). This estimate corresponds to approximately a 1% higher prevalence of self-reported AI use for each additional hour of daily digital-device use.

Perception regarding the use of AI for treatment planning also remained associated with the outcome in the adjusted model (overall *p* = 0.030). Compared with participants who disagreed that AI could be used for treatment planning, those who agreed had a 13% higher prevalence of self-reported AI use (aPR = 1.13; 95% CI: 1.00–1.28; *p* = 0.046). No statistically significant difference was observed among participants who responded “do not know” (aPR = 1.04; 95% CI: 0.90–1.20; *p* = 0.621). The overall model was statistically significant (Wald *χ*^2^(3) = 9.89; *p* = 0.020) (Table 4).

**Table 4:** Exploratory multivariable Poisson regression of characteristics associated with self-reported artificial intelligence use.

Variable	aPR (95% CI)	*p* value	Overall *p*
Daily digital-device use, per additional hour	1.01 (1.00–1.02)	0.020	
AI can be used for treatment planning			
Disagree	Ref.		0.030
Do not know	1.04 (0.90–1.20)	0.621	
Agree	1.13 (1.00–1.28)	0.046	

aPR, adjusted prevalence ratio; CI, confidence interval; AI, artificial intelligence; Ref., reference category. Adjusted prevalence ratios and 95% confidence intervals were estimated using Poisson regression with robust variance. The model included daily digital-device use and perception regarding the usefulness of AI for treatment planning. The overall *p* value for the perception variable was obtained using a post-estimation Wald test. The model was statistically significant overall (Wald *χ*^2^(3) = 9.89; *p* = 0.020). Analyses were exploratory.

In the bootstrap sensitivity analysis, estimates were highly consistent with the robust Poisson model. For daily digital-device use, the aPR was 1.010 with a bootstrap 95% CI of 1.002–1.019. For agreement that AI could be useful for treatment planning, the aPR was 1.134 with a bootstrap 95% CI of 1.013–1.309.

## 4 Discussion

The present study found a high prevalence of self-reported AI use among undergraduate medical students at a Peruvian university, with 92.45% of participants reporting previous use. Most students also self-reported basic knowledge of AI, awareness of its application in medicine, and generally favorable perceptions toward its potential role in medical practice and education. In the exploratory adjusted analysis, greater daily digital-device use and agreement that AI could be useful for treatment planning were associated with self-reported AI use. However, the magnitude of these associations was modest, and the results should be interpreted cautiously given the high prevalence of the outcome and the relatively small number of non-users.

The high prevalence of AI use observed in this study is consistent with the increasing incorporation of generative AI tools into medical education. Previous studies have reported growing use of ChatGPT and other generative AI systems by medical students for information retrieval, summarization, academic writing, brainstorming, and self-directed learning (Ganjavi et al., 2024; Weidener and Fischer, 2024; Alkhaaldi et al., 2023; Salehi et al., 2025; Abdelhafiz et al., 2025). In our study, ChatGPT was also the most frequently reported tool among the predefined options. This pattern is consistent with evidence suggesting that generative AI has rapidly become part of students’ academic practices, often preceding the development of formal curricular guidance or institutional policies governing its use (Al-Qerem et al., 2023; Ganjavi et al., 2024; Triola and Rodman, 2025; Shishehgar et al., 2025).

Because ChatGPT was the most frequently reported AI tool in this sample, the reliability of AI-generated medical content is particularly relevant. A 2025 evaluation of ChatGPT-3.5 and ChatGPT-4 on core cardiovascular physiology questions found higher accuracy for ChatGPT-4 but imperfect performance for both models and recommended faculty review of generated explanations (Banerjee et al., 2025). Thus, frequent use of an AI tool should not be interpreted as evidence that its outputs are consistently accurate or educationally appropriate.

Daily digital-device use remained associated with self-reported AI use in the adjusted analysis. Each additional hour of daily device use was associated with an approximately 1% higher prevalence of AI use. Although statistically significant, the magnitude of this association was small and its practical relevance should therefore be interpreted cautiously. Previous studies have suggested that familiarity with digital environments, technological exposure, and experience with digital platforms may be related to students’ engagement with AI technologies (Weidener and Fischer, 2024; Abdelhafiz et al., 2025; Jackson et al., 2024). Nevertheless, because the present study was cross-sectional, the temporal direction of this association cannot be established. Greater digital exposure may facilitate opportunities to encounter and use AI tools, but it is also possible that students who use AI more frequently spend more time using digital devices.

Perceptions regarding the usefulness of AI for treatment planning were also associated with self-reported AI use in the exploratory adjusted model. Students who agreed that AI could be used for treatment planning had a higher prevalence of AI use than those who disagreed. This finding is consistent with previous studies showing that medical students often express favorable perceptions toward the potential usefulness of AI in clinical practice and medical education (Weidener and Fischer, 2024; Bautista Huaytalla and Flores Larzo, 2024; Abdelhafiz et al., 2025; Jackson et al., 2024; Tajima et al., 2025). Because exposure and perception were measured at the same time, we cannot determine whether favorable views of treatment-planning applications preceded AI use or developed after students had already used these tools. The observed association may therefore partly reflect that existing AI users perceive AI as useful rather than a factor that precedes subsequent adoption.

Importantly, not all favorable perceptions were associated with AI use after the regression analyses were revised. Although a large proportion of participants believed that AI would contribute to major advances in medicine and described its use as exciting, these perception items did not show statistically significant overall associations with the outcome in the bivariate analysis. This distinction is relevant because favorable attitudes toward AI should not be assumed to correspond directly to its use. Previous research has similarly shown that awareness, attitudes, perceived usefulness, and actual engagement with AI may not develop uniformly among medical students (Bisdas et al., 2021; Civaner et al., 2022; Weidener and Fischer, 2024; Jackson et al., 2024).

The overall perception profile in our study was favorable but also reflected caution regarding some clinical applications. Most participants agreed that AI could contribute to major advances in medicine and supported its incorporation into undergraduate and postgraduate education, whereas most disagreed that AI would replace physicians. More than half also disagreed that AI should be considered a definitive diagnostic tool. These findings are consistent with studies in which medical students tend to view AI as a complementary resource rather than a substitute for professional judgment and clinical reasoning (Bisdas et al., 2021; Weidener and Fischer, 2024; Jackson et al., 2024). Concerns regarding hallucinations, bias, transparency, privacy, accountability, and inappropriate reliance on AI-generated information further support the importance of maintaining human oversight when these technologies are introduced into medical education and future clinical practice (Triola and Rodman, 2025; D'Souza et al., 2024; Li et al., 2025; Itani et al., 2025).

The high prevalence of AI use should also not be interpreted as evidence of adequate AI competence. In this study, basic AI knowledge was self-reported rather than objectively assessed. Therefore, the high proportion of students reporting basic knowledge may reflect perceived familiarity rather than demonstrated AI literacy. Evidence from medical students and practicing physicians suggests that awareness, familiarity, practical use, critical appraisal, and understanding of limitations may not develop in parallel (Weidener and Fischer, 2024; Banerjee et al., 2024; Shishehgar et al., 2025; D'Souza et al., 2024; Li et al., 2025). AI literacy should therefore not be treated as a single construct, and medical education should distinguish routine tool use from the competencies required to critically evaluate AI-generated information, recognize bias and limitations, and apply appropriate ethical and clinical boundaries.

These findings have implications for undergraduate medical education. Given the widespread self-reported use of AI in this population, educational strategies may need to move beyond simply introducing students to AI tools. Structured training should emphasize critical appraisal of AI-generated outputs, ethical reasoning, data privacy, academic integrity, bias recognition, transparency, and appropriate limits of AI use. Current literature increasingly emphasizes that the integration of AI into medical education requires not only technical knowledge but also institutional governance, faculty development, and clear educational policies (Triola and Rodman, 2025; Shishehgar et al., 2025; D'Souza et al., 2024; Li et al., 2025; Itani et al., 2025). Such approaches may help align students’ rapidly evolving use of AI with principles of responsible and evidence-informed medical practice.

These considerations may be particularly relevant in settings where access to publicly available AI tools develops more rapidly than formal educational or institutional guidance. Local evidence is therefore important because patterns of technology access, institutional support, digital literacy, and educational needs may differ across contexts. The present study provides data from a Peruvian medical school, complementing the still limited local evidence on medical students’ perceptions and use of AI (Bautista Huaytalla and Flores Larzo, 2024). However, these findings should not be assumed to represent medical students throughout Peru or other educational settings.

This study has several limitations. First, its cross-sectional design precludes establishing temporality or causal relationships. In particular, the observed associations between digital-device use, perceptions regarding treatment planning, and AI use may be subject to reverse causality. Second, the use of non-probability convenience sampling and recruitment through WhatsApp groups may have preferentially reached students who were more digitally engaged, more interested in AI, or more likely to respond to an online survey. Because the number of students who received or viewed the invitation was not recorded, a formal response rate could not be calculated, limiting assessment of sample representativeness. Consequently, the observed 92.45% prevalence should be interpreted as the prevalence among surveyed respondents rather than as a population-level estimate for all medical students at the university or in Peru. Third, the study was conducted at a single university, further limiting generalizability to other medical schools or student populations.

In addition, the outcome was defined broadly as having previously used an AI tool. This definition did not distinguish between occasional and frequent use, intensity of use, or dependence on AI tools and therefore had limited discriminatory capacity because most participants reported AI use. Only 21 participants were classified as non-users, which restricted the information available for regression modeling and increased the potential for sparse-data bias and overfitting. To reduce this risk, the adjusted analysis was intentionally restricted to a parsimonious exploratory model, and the resulting associations should be interpreted cautiously rather than as definitive independent predictors.

Additional limitations relate to measurement. AI knowledge was self-reported rather than objectively tested, and some listed platforms, such as Canva and Grammarly, contain both conventional and AI-enabled functionalities. Because the questionnaire did not identify the specific functionality used within these platforms, some misclassification is possible. Furthermore, the response format of the perception instrument was adapted from five to three categories before data collection. Although the adapted instrument showed adequate internal consistency and sampling adequacy for factor analysis, modifications to the original response structure may affect comparability with studies using the original instrument. Finally, all information was self-reported and may therefore be subject to recall and information bias.

Despite these limitations, this study provides local evidence on the widespread self-reported use of AI among medical students and highlights the importance of distinguishing actual use from perceptions and self-reported knowledge. The findings may help inform future multicenter studies using more discriminative measures of AI use, including frequency, intensity, purposes of use, and objective measures of AI literacy.

## 5 Conclusion

Self-reported AI use for academic activities was highly prevalent among undergraduate medical students surveyed at this Peruvian university. The principal contribution of this study is the description of patterns of AI use, self-reported knowledge, and perceptions in this population.

Secondary exploratory analyses identified associations between self-reported AI use, daily digital-device use, and perceptions regarding treatment planning. These estimates should be interpreted cautiously because of the high prevalence of the outcome, the small number of non-users, and the cross-sectional design, and they should not be interpreted as predictors of AI adoption.

The findings support the need for structured educational strategies that promote critical, ethical, and responsible AI use in undergraduate medical education. Future multicenter studies should incorporate more discriminative measures of AI engagement, including frequency, intensity, purposes of use, and objective or multidimensional measures of AI literacy.

## Data Availability

The datasets presented in this study can be found in online repositories. The names of the repository/repositories and accession number(s) can be found at: https://docs.google.com/spreadsheets/d/19c16a4DSPI3oNprOlLVCmL0OHpfly7UG/edit?usp=sharing&ouid=117537439755233653908&rtpof=true&sd=true.

## Supplementary material

Supplementary Table S1 Promax-rotated factor loadings of the adapted 14-item AI perception scale.
