# Supplementary material for: Self-reported artificial intelligence use, knowledge, and perceptions among undergraduate medical students at a Peruvian university
Source: Front Artif Intell. 2026 Oct 29;9:1940222. doi: 10.3389/frai.2026.1940222 (PMC13755248; doi:10.3389/frai.2026.1940222)
Supplement: Supplementary file 1 [file Table_1.DOCX]

**SUPPLEMENTARY TABLE**

**Supplementary Table S1. Promax-rotated factor loadings of the adapted 14-item AI perception scale**

| **Items** | **Clinical applications of AI** | **General attitudes and educational integration** | **Uniqueness** |
| --- | --- | --- | --- |
| AI will lead to major advances in medicine | - | 0.607 | 0.633 |
| AI will replace physicians in the future | 0.398 | - | 0.895 |
| AI can be used as a definitive diagnostic tool | 0.564 | - | 0.715 |
| AI can be used as a prognostic tool | 0.347 | 0.416 | 0.538 |
| AI can be used for treatment planning | 0.518 | - | 0.532 |
| AI can be used for treatment quality control | 0.422 | 0.345 | 0.532 |
| AI applications should be part of undergraduate training | - | 0.792 | 0.346 |
| AI applications should be part of postgraduate training | - | 0.771 | 0.349 |
| The use of AI in medicine is exciting | - | 0.818 | 0.392 |
| AI can be used for radiographic diagnosis | 0.855 | - | 0.219 |
| AI can be used for lesion diagnosis | 0.849 | - | 0.286 |
| AI can be used for radiographic diagnosis of respiratory pathologies | 0.878 | - | 0.212 |
| AI can be used for radiographic diagnosis of intestinal obstruction | 0.957 | - | 0.154 |
| AI can be used in forensic medicine | 0.710 | - | 0.388 |

Note: Exploratory factor analysis was conducted using a polychoric correlation matrix and iterated principal-factor extraction, followed by oblique Promax rotation. Absolute factor loadings <0.30 are suppressed. The correlation between the two factors was 0.587. Cronbach’s α for the complete scale was 0.8699 and McDonald’s ω was 0.933. *The item concerning replacement of physicians was reverse-scored for the psychometric analysis.
